# Electronic Circular Dichroism Detects Conformational Changes Associated with Proteasome Gating Confirmed Using AFM Imaging

**DOI:** 10.3390/biom13040704

**Published:** 2023-04-20

**Authors:** Alessandro D’Urso, Roberto Purrello, Alessandra Cunsolo, Danilo Milardi, Caterina Fattorusso, Marco Persico, Maria Gaczynska, Pawel A. Osmulski, Anna Maria Santoro

**Affiliations:** 1 Dipartimento Scienze Chimiche, Università degli Studi di Catania, Viale A. Doria 6, 95125 Catania, Italy; rpurrello@unict.it (R.P.); alessandracunsolo86@gmail.com (A.C.); 2 Istituto di Cristallografia—CNR Sede Secondaria di Catania, Via P. Gaifami 18, 95126 Catania, Italy; danilo.milardi@ic.cnr.it; 3 Dipartimento di Farmacia, Università di Napoli “Federico II”, Via D. Montesano 49, 80131 Napoli, Italy; caterina.fattorusso@unina.it (C.F.); m.persico@unina.it (M.P.); 4 Department of Molecular Medicine, University of Texas Health at San Antonio, San Antonio, TX 78229, USA; gaczynska@uthscsa.edu (M.G.); osmulski@uthscsa.edu (P.A.O.)

**Keywords:** 20S proteasome, cationic porphyrins, (AFM) atomic force microscopy imaging, allostery, electronic circular dichroism (ECD)

## Abstract

Many chronic diseases, including cancer and neurodegeneration, are linked to proteasome dysregulation. Proteasome activity, essential for maintaining proteostasis in a cell, is controlled by the gating mechanism and its underlying conformational transitions. Thus, developing effective methods to detect gate-related specific proteasome conformations could be a significant contribution to rational drug design. Since the structural analysis suggests that gate opening is associated with a decrease in the content of α-helices and β-sheets and an increase in random coil structures, we decided to explore the application of electronic circular dichroism (ECD) in the UV region to monitor the proteasome gating. A comparison of ECD spectra of wild type yeast 20S proteasome (predominantly closed) and an open-gate mutant (α3ΔN) revealed an increased intensity in the ECD band at 220 nm, which suggests increased contents of random coil and β-turn structures. This observation was further supported by evaluating ECD spectra of human 20S treated with low concentration of SDS, known as a gate-opening reagent. Next, to evaluate the power of ECD to probe a ligand-induced gate status, we treated the proteasome with H2T4, a tetracationic porphyrin that we showed previously to induce large-scale protein conformational changes upon binding to h20S. H2T4 caused a significant increase in the ECD band at 220 nm, interpreted as an induced opening of the 20S gate. In parallel, we imaged the gate-harboring alpha ring of the 20S with AFM, a technique that we used previously to visualize the predominantly closed gate in latent human or yeast 20S and the open gate in α3ΔN mutant. The results were convergent with the ECD data and showed a marked decrease in the content of closed-gate conformation in the H2T4-treated h20S. Our findings provide compelling support for the use of ECD measurements to conveniently monitor proteasome conformational changes related to gating phenomena. We predict that the observed association of spectroscopic and structural results will help with efficient design and characterization of exogenous proteasome regulators.

## 1. Introduction

Proteasome is a supramolecular machine acting as central keeper of the proteostatic balance with a vital importance for nearly all aspects of cellular metabolism [1]. Consequently, proteasome dysfunctions profoundly influence the wellbeing of organisms and are associated with numerous human pathologies. Thus, an understanding of proteasome regulation is critically relevant to almost all facets of human health [2,3].

Proteasome comprehends several multi-subunit assemblies that share the catalytic core particle 20S; the latter can work stand-alone (“naked” form) [4] or complexed with a plethora of regulatory particles (RPs: PA700 (19S), PA28 (11S) and PA200) [5] and is regulated by other interacting proteins, such as IDE [6,7] and chaperones [8]. The most advanced RP, the 19S, is involved in substrate recognition, deubiquitination, unfolding and translocation into the catalytic core [9]. Depending whether the 20S is complexed with one or with two 19S units, the assemblies are termed 26S or 30S, respectively [10].

As an adaptive response to cellular needs, in stress condition and in the presence of oxidatively damaged proteins, the 26S disassembles and the uncapped 20S core becomes the most abundant (up to 80%) particle [11]. The core is engaged in ubiquitin-independent degradation, which represents the preferential pathway for intrinsically disordered proteins (IDPs) [12].

Structurally, the 20S catalytic core particle (CP) is tube-shaped and consists of 28 subunits, which are arranged in a stack of four heptameric rings in an αββα fashion. The catalytically active sites are located inside the core particle at subunits β1, β2, and β5 and have, respectively, caspase-like (also named PGPH; post-glutamyl peptide hydrolyzing), trypsin-like (post-basic residues cleavage; T-L), and chymotrypsin-like activities (post-hydrophobic residues cleavage; ChT-L) [13]. The substrate access to the catalytic chamber is controlled by a gate located at the center of the two outer α rings and consisting of tightly packed *N*-termini of all α-subunits [14]. Upon binding of the RP, these *N*-termini are destabilized which results in opening the gate and shifting the 20S core from the latent (closed) to the processing (open) state [15]. On the other hand, in the naked form (prevalent under stressful conditions), the 20S exists in equilibrium between different conformational states, among which the closed-gate state prevails (~75%), [16]. Indeed, gating is essential for regulation of the proteasomal activity in the cell [17,18,19].

The 20S proteasomes are highly dynamic and allosterically regulated by an intricate array of signals. Such dynamics allows these molecules to display multiple conformations with different activities [20]. In fact, besides the two macrostates exemplified as “open” and “closed”, there is a multitude of intermediate states that finely regulate the access of substrates and efficiency of degradation [21,22,23]. These “conformation populations” or “ensembles” have been experimentally explored using CryoEM [24], AFM microscopy [25], and NMR [26].

Here, taking into account the relative abundance of α-helix, β-sheets, and random-coils present in experimentally determined structures of the “open” and “closed” 20S conformations, we show that ECD spectroscopy can be used to sense the conformational changes of the 20S proteasome associated with the gating mechanism. Moreover, we probed power of this approach by correlating ECD with AFM based observations using the cationic porphyrin H2T4.

**Scheme 1:**
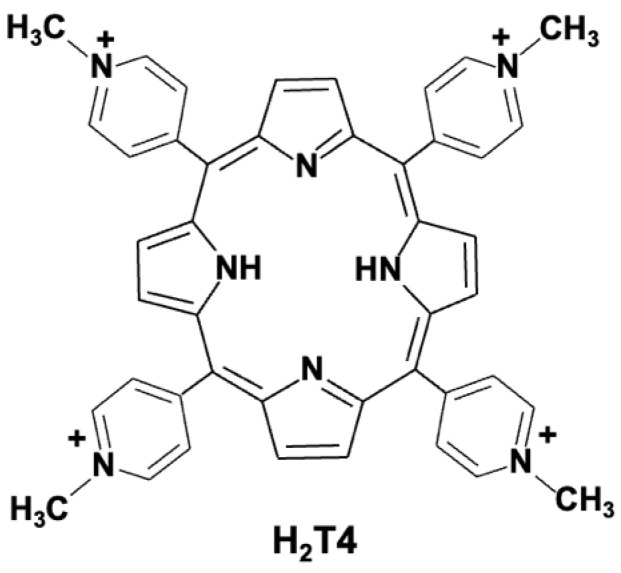
Structure of 5,10,15,20-tetrakis-4-*N*-methylpirydyl porphyrin (H2T4).

We previously proposed that certain cationic porphyrins modulate proteasome activity by exploiting the “electrostatic code” involved in the binding to RPs [22,27]. In that respect, the tetra-cationic porphyrin H2T4 (Scheme 1), presented the best pharmacophore fit with the four aspartate residues located at the gate entrance and a second putative binding site at the interface between the 20S α and β subunits [28] partially overlapping with the binding site of chloroquine on *Thermoplasma acidophilum* 20S [28,29].

On the other hand, kinetic investigation using stopped-flow UV spectroscopy pointed at a large-scale conformational change of the protein upon H2T4 binding, proceeding with a fast interaction step followed by a slow step. We next demonstrated that the ability of H2T4 to induce large-scale conformational changes in the core proteasome molecule represents a common feature with the other active porphyrins acting as positive or negative allosteric modulators of the 20S. All these 20S ligands share the ability to affect gating dynamics and, by consequence, the relative populations of multiple and interconverting 20S conformations [22,27,28].

Surprisingly, even if ECD is widely used to study of protein structure in the UV region [30], where the marker bands of the various structural motifs are found, no ECD studies of the proteasome have been reported till now. It is worth to note that the ECD of polypeptides does not merely represent the optical activity of a single specific chromophore (the amide in our case), but it provides information on the coupling between chromophores as modulated by their reciprocal spatial arrangement. The latter modulates not only the sign and shape of the ECD typical of different limit conformations (see for example the ECD of left- and right-handed helix or that of coil and helix), but also the change of the signal intensity due to subtle variations which do not lead to drastic conformational switches, but only to a different dipole–dipole coupling. This means that even in the absence of net conformational changes the intensity of the various marker bands may vary. Therefore, we considered ECD to be a suitable tool to detect ligand-induced large scale conformational changes in the 20S molecules. Modulation of the state of proteasomal gate by H2T4 is verified here using a combined approach in which computational, ECD and AFM studies converge in an attempt to represent this complex system.

## 2. Materials and Methods

### 2.1. Secondary Structure Analysis

The following experimentally determined structures of human (h20S) and yeast (y20S) 20S proteasome were downloaded from the Protein Data Bank (PDB, http://www.rcsb.org/pdb/, accessed on 31 January 2023): (i) native h20S proteasome (closed state; PDB ID: 5LE5) [31], (ii) h20S in complex with 19S (open state; PDB ID: 6MSK) [24], (iii) native y20S proteasome (closed state; PDB ID: 1RYP) [32], and (iv) fully open mutant α3ΔN y20S (PDB ID: 1G0U) [33]. Then, secondary structure analysis was performed using the server “2Struc: The Secondary Structure Server” [34] by selecting four different methods: (i) Dictionary of Secondary Structure of Proteins (DSSP) [35] (ii) DSSPcont [36] (iii) STRuctural IDEntification (STRIDE) [37] and (iv) XTLSSTR [38]. The DSSP method assigns eight states of secondary structure based solely on hydrogen bonds. Based on a Coulomb approximation of the hydrogen bond energy, DSSP defines a hydrogen bond as having a bond energy of less than −0.5 kcal/mol. DSSPcont is a DSSP variant that aims to assign secondary structure in a continuous manner, reflecting structural variability caused by thermal motion. The continuous output is obtained by running DSSP multiple times with different hydrogen bond thresholds and then imposing a weighted average over each residue’s individual DSSP assignments.

The STRIDE method [37] assigns secondary structure based on empirically derived hydrogen bond energy and phi–psi torsion angles. Torsion angle propensities are assigned based on how close they are to their respective regions in the Ramachandran plot. STRIDE has been optimized for the X-ray assignments. Finally, the XTLSSTR method assigns secondary structures using distances and angles calculated from backbone geometry. It was designed to match comparable data determined from circular dichroism spectra by deriving secondary structure from three-dimensional coordinates.

### 2.2. Electronic Circular Dichroism (ECD) Experiments

ECD spectra were recorded at 22 °C using a Jasco J-715 spectropolarimeter equipped with a single position Peltier temperature control system. A quartz cuvette with a 0.1 cm path length was used for all ECD experiments. Conditions were as follows: scanning rate 20 nm/min, data pitch 0.2 nm, digital integration time (D.I.T) 4 s, band width 2.0 nm. Each ECD spectrum was an average of at least ten scans. Experiments were carried out by diluting h20S (up to a final 25 nM concentration) in 150 mM Tris-HCl + 0.1 M NaCl at pH 7.6. Each ECD experiment was ran in triplicate. For each ECD, we then subtracted the “reference” system spectrum and obtained three difference ECD, which, in all cases, are almost superimposable.

### 2.3. Atomic Force Microscopy (AFM) Imaging

We used the established procedure of single-molecule imaging of live proteasome particles in liquid with tapping (oscillating) AFM mode, as previously described [39,40]. In short, the 20S proteasome (Enzo Life Sciences Inc.; Farmingdale, NY, USA) was electrostatically immobilized on muscovite mica (Ted Pella Inc.; Redding, CA, USA) and covered with 5 mM Tris–HCl buffer, pH 7 (the imaging buffer). Scanning and imaging were performed with SNL (Sharp Nitride Lever) probes with cantilevers of 0.35 N/m spring constants, with a scanner E of the Multimode Nanoscope IIIa (probes and instrument: Bruker Inc., Santa Barbara, CA, USA). The amplitude setpoint was in the range of 1.4–1.8 V, drive voltage of 200–500 mV, and scanning rate of 2.63 or 3.05 Hz. Scans of 1 µm × 1 µm fields, trace and retrace, were collected in the height mode with a digital resolution of 512 × 512 pixels. Multiple fields were scanned to collect images of hundreds of proteasome particles; the majority of them imaged in the top-view position with the α face exposed. Selected fields were repeatedly scanned at least four times (at least 12 min) to monitor changes in the topography of the same particles [41]. To follow conformational response of proteasomes, core particles after settling on mica were scanned first (“controls”), with images of at least four fields collected. Then, 10 µL of H2T4 dissolved in imaging buffer was injected directly into the wet chamber to the final concentration to 0.2 µM and the scanning resumed. To test the response of control proteasomes or H2T4–treated proteasomes to the model peptide substrate, the solution of SucLLVY–MCA (the model substrate for the leading ChT-L proteasome peptidase) was injected to the chamber to the final concentration of 100 µM, and the scanning continued. Neither injection process nor the presence of 1% DMSO (solvent for the SucLLVY-MCA) affected topography of the proteasomes, as was demonstrated previously [40]. The presented images of top-view core particles are raw, with a standard order-one plane-fit and flattening [NanoScope software version 5.12; Scanning Probe Image Processor (SPIP) version 6.0.13; Image Metrology, Hørsholm, Denmark] used as processing tools. Classification of the conformation of α face of a single top-view particle was carried out, as previously described, by analyzing numerical values of the height of pixels in scan-lines crossing the area of the gate in the center of the α face [23,25,39]. We distinguished three conformational states of the α face and the gate: closed (the plot of height values in the scan-line presented a convex function), intermediate (a concave function without a local minimum) and open (a central dip or, in other words, a local minimum detected). The data presented are averages of percent partition of the three conformers in at least three fields in at least two independent experiments, with about 50–100 particles per field analyzed.

## 3. Results and Discussion

### 3.1. Structural Analysis

Before engaging in the ECD and AFM experiments, we performed the analysis of the secondary structure composition of the 28 protein subunits of h20S in the closed and open-gate state. We found that the gate opening is associated with significant structural changes in the secondary structure composition.

**Figure 1:**
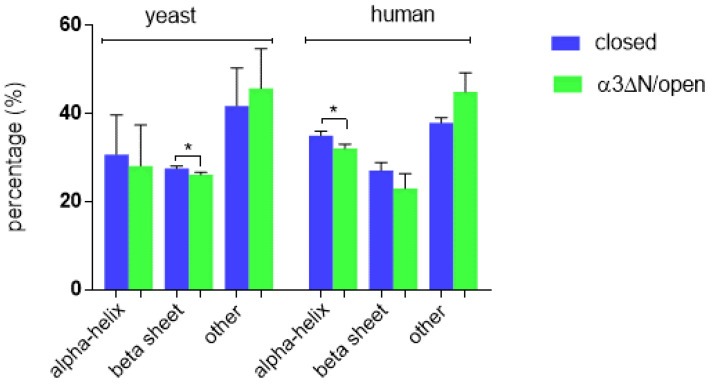
Comparison of secondary structure content in yeast (left) and human (right) 20S proteasomes in putative open- and closed-gate conformations. The column graph shows the means (+/− SEM) of percentages of alpha-helix, beta-sheet, and other structural motifs in closed (blue columns) and open proteasome (green columns) calculated using four different methods (DSSP, DSSPcont, XTLSSTR and STRIDE) using the 3D structures of the enzymes. PDB codes: 5LE5 for closed h20S, 6MSK for open h20S; 1RYP for closed y20S; 1GOU for the fully open-gate mutant α3ΔN y20S that lacks the gate-closing *N*-terminal fragment of the α3 subunit. Analysis of significance has been performed with *t*-test (* *p* < 0.05).

The analysis of the secondary structure of the closed and open-gate states of h20S proteasome was carried out using four different methods selected to provide a robust structure assessment [34]. Each method is based on a distinct algorithm and, accordingly, the outcomes vary both in the calculated parameters, i.e., hydrogen bond patterns and/or backbone torsion angles and distances, and in how these parameters are taken into account in the identification of the secondary structure elements (α-helix and β-sheet; see Section 2 for details). In particular, two methods, DSSP [35] and DSSPcont [36], were used calculate the hydrogen bond patterns. The DSSPcont performs the calculations multiple times with different hydrogen bond thresholds thus taking into account structural variability caused by thermal motion. In our case, the two methods converged in nearly identical sets of values (Table 1, Figure 1). The other two methods derive a secondary structure from three-dimensional coordinates and were suited for matching data generated with the help of circular dichroism spectroscopy for XTLSSTR [38] and X-ray crystallography for STRIDE [37]. The latter uses stringent filters for determining the presence of secondary structure elements, and, accordingly, it showed the lowest rate of organized secondary structure compared to the other methods (Table 1 and Figure 1).

The data indicate that the transition from closed to the open state is accompanied by a lower participation of α-helix and β-sheet (changes in the range of 1.6–5.2% for α-helix and 1.3–5.6% for β-sheet), while the contribution of “other secondary structures” increases (changes in the range of 3.0% to 10.8%).

### 3.2. ECD Studies

First, we attempted to explore if the gate movements are accompanied by variations of the ECD spectra robust enough to allow for distinction between the open and closed-gate states. For this purpose, we compared the ECD spectra of the mainly “closed” resting (latent) states of yeast and human 20S (namely, y20SWT and h20S) with those of the “open” states of the yeast mutant blocked in an open conformation of the gate (yα3ΔN) and h20S treated with SDS (detergent inducing gate opening), [42], respectively.

**Figure 2:**
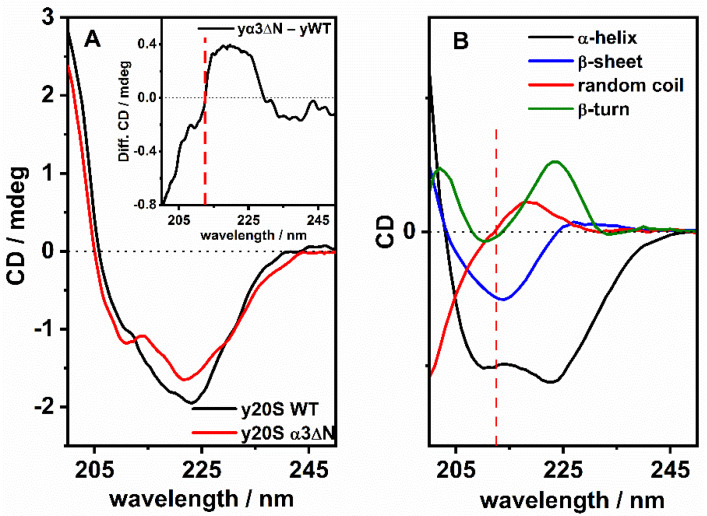
(**A**) Comparison between ECD spectra of y20S WT 25 nM, in 150 mM Tris buffer with 100 mM of NaCl at pH 7.6 (black trace) and y20S α3∆N mutant in the same buffer (red trace). Inset: ECD difference between the spectra of α3∆N minus that of WT. (**B**) ECD spectra of canonical conformations of polypeptides. From here after, the red dotted line indicates the cross-over wavelength (the point where ECD change sign) of the ECD signal for random coil.

Interestingly, the ECD spectrum of yα3ΔN was markedly different from that of y20SWT (Figure 2A). In particular, the ECD spectrum of the mutant shows that the negative band at 208 nm is more intense than that of y20SWT while the intensity of the broad band centered at about 222 nm is lower in the mutant.

In order to visualize these differences, we subtracted the spectra of α3∆N and WT proteasomes (i.e., particles with an open gate minus those with a closed gate). The resulting ECD difference spectrum (inset in Figure 2A) clearly shows that ECD is sensitive to the conformational changes of proteasome accompanying the opening of the gate. In particular, the spectrum shows a negative band below 210 nm and a positive band centered at around 225 nm (inset in Figure 2A).

To aid interpretation of the difference spectrum, we provide in Figure 2B the theoretical ECD spectra of canonical pure structural motifs of a generic polypeptide. It is worth to point out that, simplifying, both α -helical an β-sheet have negative signals (two for the helices and one for the strands) in the region around 220 nm and a positive band below about 200 nm. The ECD of the two conformations differ also for the position of the point of cross over that for the α-helix is blue shifted (about 200 nm) with respect to β-sheet (in the 210 nm region). The random coil (RC) is characterized by a positive contribution at about 220 nm, a negative band at about 200 nm and a cross over point in the 210 nm region. A positive contribution at 225 nm is also observed for β-turns, together with a positive signal in the 200 nm region.

The difference “open-closed” ECD spectrum (inset of Figure 2A) resembles that observed for the RC (Figure 2B): it shows a positive band spanning from 210 nm to 235 nm, broader than that assigned to a random coil conformation and centered at 225 nm, plus a negative band below 210 nm. The broad positive feature of the difference spectrum suggests that the content of β-turns may increase during gate opening. Therefore, we may speculate that the conformational changes accompanying the path from the closed to the open state of the gate involve an increase content of “random coil” and β-turn, the only two structures that show a positive band in the region of 220 nm (Figure 2B). Therefore, our spectroscopic data are in line with the results of structural analysis shown in Table 1 that foresee a decrease in the α-helical and β-sheet in favor of other structures. It is worth to note that the ECD difference spectrum reports all the conformational changes and therefore it could be possible that the broad positive band obscures a decrease in the 222 nm band of the α-helix (that in difference spectrum is revealed as a positive contribution) forecasted by the data in Table 1.

**Figure 3:**
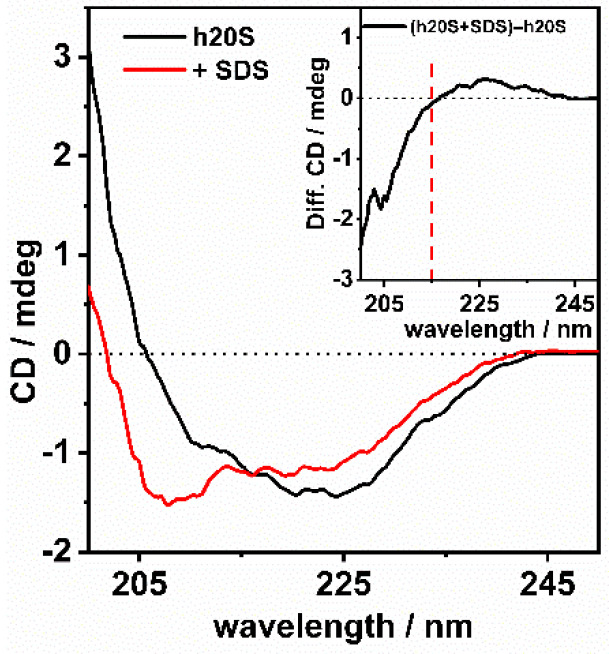
ECD spectra of 25 nM h20S proteasome in 150 mM Tris buffer with 100 mM NaCl at pH 7.6, before (black trace) and after addition of SDS 0.018% (red trace). Inset: ECD difference between the spectra of h20S in the presence of SDS minus that of h20S.

Finally, we set to compare the spectra of latent, predominantly closed h20S with h20S treated with a low concentration of the SDS detergent. Since the enhancement of the proteasome activity is related to the 20S gate opening, and SDS is known as the artificial activator of proteasome, it is reasonable to expect that the treatment with SDS would induce conformational changes towards the opening of 20S gate [42]. Not surprisingly, as shown in Figure 3, the ECD spectrum of human 20S changes strikingly after the addition of SDS. Again, the difference spectrum (inset in Figure 3) bears the distinctive signature of the increased RC content (compare with Figure 2B).

Importantly, as suggested by ECD data, both human and yeast 20S proteasomes seem to share the secondary-structure changes associated with gate opening.

Encouraged by the results described in Figure 2 and Figure 3 we decided to study the interactions of H2T4 (Scheme 1) with 20S. Since the H2T4 ligand does not absorb in the investigated UV region and consistently with its achiral structure is ECD silent, its ECD spectrum of H2T4 in solution is featureless (not shown). Therefore, we hypothesized that ECD would be able to detect ligand-induced conformational changes in the 20S proteasome, in particular changes relevant to the shifts in the equilibrium of populations with the closed or open gate.

**Figure 4:**
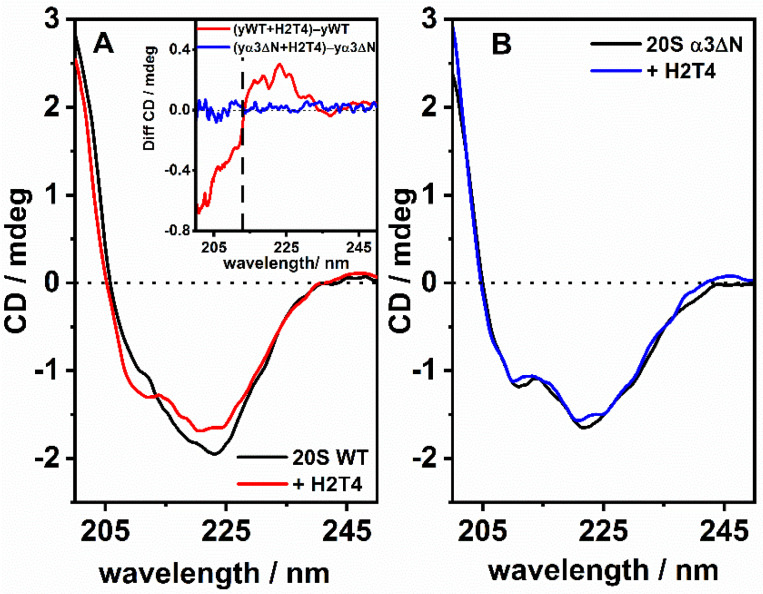
ECD spectra of y20S (panel **A**) and yα3ΔN (panel **B**) 20S proteasomes 25 nM, in 150 mM Tris buffer with 100 mM of NaCl at pH 7.6, before (black trace) and after addition of H2T4 10 µM (red and blue traces for WT and yα3ΔN, respectively). Inset: comparison of ECD differences between the spectra of yα3∆N in the presence of H2T4 and that of yα3ΔN (blue traces) and yWT in the presence of H2T4 and that of yWT.

First, we recorded spectra of the yWT20S, control and treated with 1 μM of H2T4 (Figure 4). Apparently, the addition of H2T4 induces changes in the ECD spectra of yWT20S that are very similar to those observed between yα3∆N and yWT20S, i.e., the increased intensity of the band at 210 nm and a slight decrease in the 222 nm band intensity. The difference spectrum [(yWT20S +H2T4)—yWT20S] (inset of Figure 4, panel A) resembles very closely that of yWT20S—yα3N (shown in the insets of Figure 2A and Figure 3), suggesting that, under these experimental conditions, H2T4 induces the opening of the gate.

In order to confirm that the changes observed after the addition of H2T4 to yWT20S can be ascribed to the opening of the gate, we added H2T4 to the mutant yα3ΔN, blocked in an open conformation. As demonstrated in Figure 4 panel B, the ECD spectrum after the addition of H2T4 to a solution of yα3ΔN 20S is almost superimposable to that of the mutant alone (see the difference spectrum in the inset of panel A of Figure 4). This observation confirms that, in contrast to that described for yWT20S, H2T4 does not induce substantial ECD-detectable structural changes in the mutant yα3ΔN.

The presented data, therefore, support a trend indicating the increase in the ECD band ascribed to the random coil structures or to β-turns when the 20S gate opens up.

**Figure 5:**
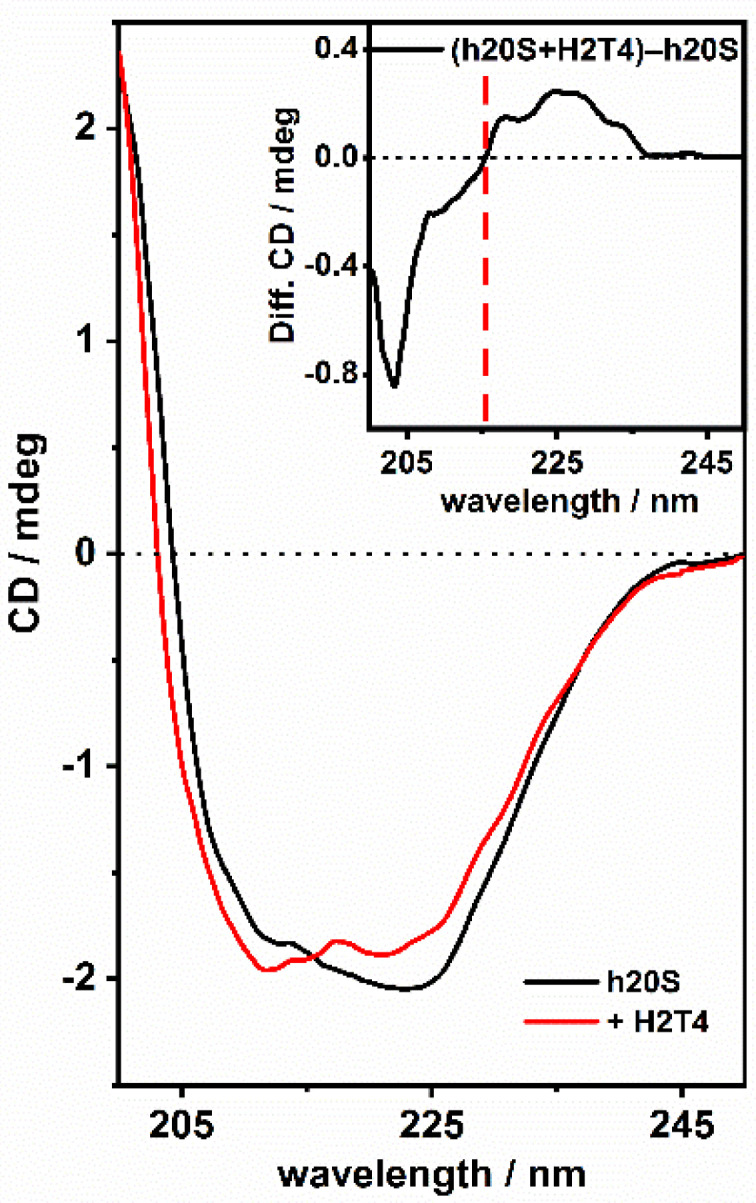
ECD spectra of 25 nM h20S proteasome in 150 mM Tris buffer with 100 mM of NaCl at pH 7.6, before (black trace) and after the addition of H2T4 1 µM (red trace). Inset: ECD difference between the spectra of h20S in the presence of H2T4 and that of h20S.

Further confirmation of this trend was obtained with ECD experiments using human 20S proteasome (h20S) (Figure 5). The addition of H2T4 to h20S induces structural shifts very similar to those reported above for yWT20S (compare Figure 4A and Figure 5). In detail, the ECD spectrum of h20S in the presence of H2T4 shows a slight increase in the intensity of the 210 nm negative band and at the same time the 222 nm negative band decreases in intensity. Indeed, the difference spectra show the positive signal at about 220 nm, the negative band at about 210 nm and the cross over at 205 nm, which we have ascribed to the conformational change accompanying the opening of the 20S gate. Consistently, the ECD data strongly suggest that the treatment with H2T4 induces opening of the gate.

Taken together the data indicate a trend of increasing participation of the random coil and/or β-turns structures when the 20S gate opens up. Specific ECD variations that indicate a putative gate opening include (i) a decrease in the band at 220 nm, which in the difference spectra is visualized as an increase in the ECD band intensity at the same wavelength and (ii) subtle variations in the region below 210 nm, that in the difference spectra give rise to a decrease in the ECD intensity in this region. However, it needs to be underlined that the difference spectra alone are not indicative of the entire scenario of conformational changes in the giant protein molecule, but only of the major structural shifts leading to the well-detectable variations in the CD spectrum.

### 3.3. Atomic Force Microscopy (AFM) Imaging

**Figure 6:**
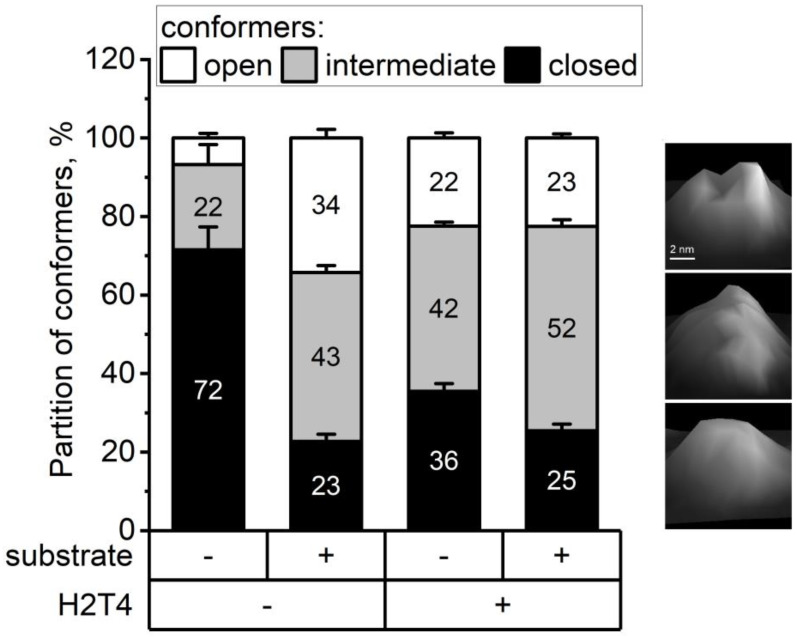
Partition of conformers in h20S proteasome detected with Atomic Force Microscopy Imaging (AFM). An addition of the model substrate or the porphyrin ligand shifted the conformational equilibrium toward less of the closed-gate forms and increased contribution of open and intermediate forms. However, an addition of the SucLLVY-MCA (succinyl-LeuLeuValTyr-7-amino-4-methylcoumarin) substrate to H2T4 (0.2 µM)—pretreated proteasomes did not result in the increase in open-gate forms, as would be expected for the substrate-processing 20S. Differences between the partitions of the same conformers in distinct cases (columns) were statistically significant in all pairs, with three exceptions: “open” (*p* = 0.95) for the H2T4 and H2T4 + substrate pair, “closed” between the substrate and H2T4 + substrate (*p* = 0.058) and “intermediate” for the control + substrate and H2T4 pair. On the right are examples of 3D projections of images of the top-view proteasomes in the three conformations, open, intermediate, closed (from the top). Averages ± SD, *n* = 3 to 7, with 211–354 particles per case analyzed. The numbers inside column segments represent the actual partitions (%).

AFM imaging allows to visualize both the open- and closed-gate populations, but also a family of intermediates states. Control “idle” proteasome particles presented the expected partition of 72 ± 6% closed, 22 ± 5% intermediate and 7 ± 1% open forms (Figure 6). Addition of the porphyrin resulted in a slow increase in the contribution of the open form, with consistent maximum readings achieved after no less than 15 min after the ligand addition. The data demonstrated in Figure 6 show only the “stable-saturated” response, with 22 ± 1.3% of open conformers. AFM results (Figure 6) show, in agreement with ECD data, that the contribution of the “open” gate population is significantly higher in H2T4—treated than in control (“idle”) proteasome particles. However, the contribution of “open” conformers does not increase after addition of the substrate to H2T4-treated proteasomes. Instead, the contribution of intermediate forms increases significantly (*p* = 0.001), from 42 ± 1.1% to 52 ± 1.7%, with these forms evidently replacing some of the closed conformers (decrease from 36 ± 1.9% to 25 ± 1.7%, *p* = 0.0024 (Figure 6).

Integrating our previous research [28] with the present results, we can hypothesize that the binding of the H2T4 to the aspartate residues of the 20S closed gate conformation (prevalent for the latent proteasome) is followed by a large-scale conformational change of the protein shifting the equilibrium toward the semi-open and open gate conformation. According to our previous docking results [28], this can be related to the reaching of a second inner binding site by H2T4, which we proposed to be located at the interface between the α and β subunits. 

## 4. Conclusions

Modulation of the 20S proteasome gating process based on the interconversion of many structurally distinct states is crucial for its functionality. Following the secondary structure analysis, we employed ECD for monitoring the equilibrium between different 20S conformational states related to gate opening/closing and used the cationic porphyrin H2T4 as a molecular probe.

Noteworthy, this is the first time that ECD has been used to investigate allosteric transitions in the proteasome treated with a small-molecule ligand. The technique proved to be remarkably sensitive to reflect the conformational changes of the gate area. Indeed, tertiary structure modifications induce variation of the coupling between the peptide transitions, which respond with intensity change of the dichroic bands in the peptide absorption region. In detail, consistent with the results of structural analysis showing a relative decrease in participation of α-helical and β-sheet structures, our ECD data suggest that the conformational changes accompanying the transition from closed to the open state of the gate involve an increase in participation of “random coil” and β-turn. Therefore, in our case, ECD efficiently detects the relationship between tertiary structures and the gate status, as observed for yWT20S and its fully open mutant form α3ΔN, both in the presence and in the absence of the tetracationic porphyrin H2T4. We found that the H2T4 behaves as a quaternary effector, inducing a conformational shift to the open conformation in the 20S proteasome through an induced-fit mechanism as a result of binding at the gate region. AFM experiments confirm this result, showing that the contribution of the “open” gate population is significantly higher in the presence of H2T4 than in control proteasome particles.

Importantly, due to the unique interactions of H2T4 with 20S, the H2T4-induced ECD changes cannot be considered universal markers for gate-opening ligands, as demonstrated by the increase in the intermediate state detected by AFM. Instead, we may hypothesize that ECD senses the decreased partition of the closed-gate conformers rather than specifically a shift toward fully open gate. In conclusion, despite the inherent limitations in the spatial resolution of ECD, this well-established and widely employed experimental technique can be added to the toolbox of methods useful for elucidating the effects of allosteric proteasome regulators. For the future, we plan to explore the association between ECD and AFM imaging outcomes to probe interactions between the proteasome and other established small-molecule or peptide-derived allosteric regulators.

## Figures and Tables

**Table 1:** Relative content of secondary structures (percentages) based on analysis of the experimentally determined structures of h20S and y20S proteasome in the closed and open conformational states.

20S Conformational State	PDB ID	Organism	Secondary Structure	DSSP ^a^	XTLSSTR	STRIDE
Closed	5LE5 (1.80 Å)	*Homo* *sapiens*	α-Helix	34.1	36.1	34.8
			β-Sheet	27.7	25.0	28.5
			Other	38.2	38.9	36.7
Open	6MSK (3.2 Å)		α-Helix	32.4	30.9	32.8
			β-Sheet	23.7	19.4	26.0
			Other	43.9	49.7	41.2
Closed	1RYP (1.90 Å)	*Saccharomyces cerevisiae*	α-Helix	35.0	36.8	20.4
			β-Sheet	27.8	26.8	27.9
			Other	37.2	36.4	51.7
Mutant/Open	1G0U (2.40 Å)		α-Helix	33.4	33.6	17.4
			β-Sheet	26.4	25.5	26.5
			Other	40.2	40.9	56.1

^a^ The same values were obtained with the DSSPcont method.

## Data Availability

The data presented in this study are available on request from the corresponding author.
